# Securing accelerated access to long‐acting injectable cabotegravir for HIV prevention in low‐ and middle‐income countries

**DOI:** 10.1002/jia2.26101

**Published:** 2023-07-13

**Authors:** Sarah Y. Jenkins, Danielle Resar, Zachary Panos, Alan Staple, Melynda Watkins, David Ripin, Carolyn Amole

**Affiliations:** ^1^ Clinton Health Access Initiative Boston Massachusetts USA

**Keywords:** cabotegravir, long‐acting, HIV prevention, access, LMICs, PrEP

## Abstract

**Introduction:**

Reductions in HIV acquisition have slowed, and the global community is significantly off track from global goals. Oral pre‐exposure prophylaxis (PrEP) alone cannot address the diverse needs of the millions of people at risk of HIV acquisition. Long‐acting injectable cabotegravir (CAB‐LA) received United States Food and Drug Administration approval for HIV prevention in December 2021. When studied, CAB‐LA demonstrated high effectiveness, provides months of protection versus daily use, is preferred by some users and has the potential to achieve commodity cost reduction. These factors position CAB‐LA to catalyse transformation in HIV prevention. Significant work must be undertaken to ensure at‐scale uptake in low‐ and middle‐income countries. Leveraging decades of product introduction experience, Clinton Health Access Initiative (CHAI) has developed an innovative roadmap to support equitable CAB‐LA introduction, comprising tightly executed market‐shaping, product development, regulatory, and programmatic and implementation action.

**Discussion:**

Proven models exist (e.g. long‐acting reversible contraceptives, paediatric tuberculosis treatment and antiretrovirals (ARVs), such as paediatric dolutegravir and tenofovir disoproxil fumarate, lamivudine, and dolutegravir) for partnership‐driven, accelerated, impactful product introduction. Based on learnings from these models and needs in the prevention space, CHAI developed a roadmap to maximize the near‐term impact of CAB‐LA and accelerate the development of, access to and impact of quality‐assured, low‐cost generic CAB‐LA. This roadmap is intended to inform introduction planning and investment decision‐making across a range of stakeholders, including donors, governments, manufacturers and other partners working in the HIV prevention space. Elements include (1) ensuring coordination and alignment across partners, and avoiding redundancy experienced during oral PrEP introduction; (2) preparing national programmes and providing support to maximize impact, including the development of national policies, guidelines and introduction plans; system strengthening; quantification and procurement; and addressing evidence needs, among other areas; (3) supporting community engagement, ensuring that demand generation and delivery approaches are person‐centred and community‐led; (4) incentivizing generic product development through, for example, milestone‐based commercialization incentives and product development cost‐sharing; and (5) expediting regulatory reviews.

**Conclusions:**

Accelerating access to affordable, generic CAB‐LA can transform progress towards HIV epidemic control. This vision of impact at scale in prevention is achievable, if informed by results‐backed approaches to introduction.

## 1 INTRODUCTION

With 1.5 million new human immunodeficiency virus (HIV) acquisition in 2021, the 2020 UNAIDS target of 500,000 annual new acquisitions was missed by a million new acquisitions [1, 2]. If trends continue, the 2025 target will be missed by over a million new acquisitions [2]. High rates of HIV acquisitions persist despite significant progress increasing treatment coverage and viral suppression. Increasing access to effective prevention interventions, including condoms, voluntary medical male circumcision (VMMC) and oral pre‐exposure prophylaxis (PrEP), will be critical to achieve and sustain epidemic control. However, there is an urgent need to expand the portfolio of prevention options to support person‐centred services aligned with user preferences and lifestyles. As demonstrated in family planning, expanded options can also drive increased uptake and coverage [3].

Oral PrEP uptake has increased, with an estimated 2.8 million cumulative initiations as of mid‐2022 and over a million new initiations in 2021 [4, 5]. However, annual initiations remain below the 2020 target of 3 million persons accessing PrEP and off track to reach the 2025 target of 10 million initiations [6, 7]. Moreover, these data do not reflect the number currently using PrEP and most programmes are unable to quantify the duration of use. Many of those at the highest risk of HIV face challenges using oral PrEP, including stigma, pill burden and low‐risk perception [8, 9, 10]. Oral PrEP alone cannot address the diverse needs of the millions at risk of HIV.

Long‐acting injectable cabotegravir (CAB‐LA) received United States Food and Drug Administration (US FDA) approval in December 2021 [11]. In clinical trials, CAB‐LA was shown to be safe and effective at preventing HIV [12, 13]. As a bimonthly injection, CAB‐LA offers a discreet, long‐acting option aligned with the preferences of many at risk of HIV [14, 15, 16, 17]. The analysis also indicates that generic manufacturer can produce CAB‐LA affordably [18]. Based on these factors, CAB‐LA has the potential to catalyse transformation in HIV prevention. However, barriers to access remain. Generic manufacture is expected to take several years and, while production costs are estimated to be low, without intervention, an affordable generic price is not guaranteed. Moreover, in the near‐term, supply is limited in a single supplier market. Pricing from ViiV Healthcare, the originator of CAB‐LA, is also expected to be substantially higher than the price of oral PrEP. Significant work is needed to enable the widespread use of CAB‐LA, particularly in low‐ and middle‐income countries (LMICs). With few countries delivering oral PrEP at scale, health systems lack experience with high‐volume PrEP delivery. Oral PrEP experience also demonstrated that poorly coordinated implementation projects can lead to persistent evidence gaps, slowing adoption and scale‐up. For example, in 2018, fewer than 21 LMICs had adopted the World Health Organization (WHO) oral PrEP guidelines [19].

Leveraging decades of product introduction experience, Clinton Health Access Initiative (CHAI) developed an innovative roadmap to accelerate equitable CAB‐LA introduction. This roadmap was informed by ongoing collaborations, including with the Coalition to Accelerate Access to Long‐Acting PrEP, convened by Unitaid, WHO, UNAIDS, Global Fund, and the US President's Emergency Plan for AIDS Relief (PEPFAR) and other PrEP planning mechanisms.

Alongside implementation considerations and research gaps outlined in WHO guidelines, partners have initiated the development of introduction plans for CAB‐LA [20, 21]. This roadmap complements those plans by leveraging an evidence‐based approach to systematically document successful introduction models and identify how approaches need to be adapted for CAB‐LA. Previously published work has taken a similar evidence‐based approach, investigating market‐shaping investments needed to bring long‐acting products, such as CAB‐LA, to the generic marketplace [22]. However, this paper addresses a more comprehensive scope of introduction needs, from the collaborations needed to facilitate market‐shaping investments to the engagement required to generate demand at the community level. It aims to inform planning and decision‐making among donors, governments and other partners working to accelerate access and impact of CAB‐LA.

## 2 DISCUSSION

Recent HIV treatment transitions demonstrated the pace and scale of innovation possible through collaborative partnerships [23]. In 2018, a breakthrough agreement between CHAI, the UK's Foreign and Commonwealth Development Office, Unitaid, Bill & Melinda Gates Foundation, the governments of South Africa and Kenya, PEPFAR, the Global Fund, WHO, UNAIDS, United States Agency for International Development (USAID), Mylan and Aurobindo to accelerate access to the fixed‐dose combination of tenofovir disoproxil fumarate, lamivudine and dolutegravir (TLD) achieved an LMIC launch price lower than the standard of care for the first time ever [24]. By 2021, TLD was included in national guidelines in over 120 countries with over 18 million patients on dolutegravir (DTG)‐based regimens in LMICs at a price of under US$50 per patient per year [25, 26]. The successful transition to TLD was driven by this landmark pricing agreement alongside effective global coordination, tireless community advocacy and engagement, intensive in‐country planning and targeted market‐shaping interventions.

There are also important models to leverage from product introduction in sexual and reproductive health (SRH), especially given similarities with the multi‐product, choice‐driven landscape. In 2013, a CHAI agreement, which lowered the price of long‐acting reversible contraception from US$18 to US$8.50 per implant and led to a five‐fold increase in consumption shows that well‐designed, timely market interventions translate into health benefits for large populations [27].

**Figure 1:**
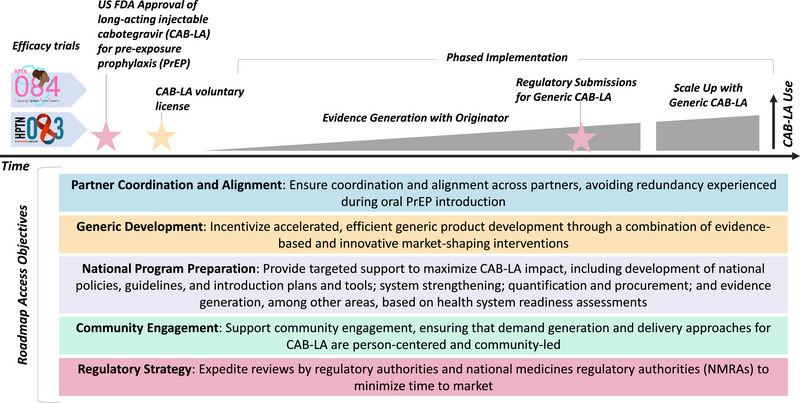
Long‐acting injectable cabotegravir (CAB‐LA) roadmap areas and access objectives.

Leveraging successful product introduction experience and learnings, this roadmap outlines a pathway to maximize impact and accelerate the development and introduction of quality‐assured, affordable generic CAB‐LA. The roadmap addresses five thematic areas outlined in Figure 1. While we focus on CAB‐LA, insights can also inform planning for other PrEP products, like the dapivirine vaginal ring.

### 2.1 Partner coordination and alignment

During TLD rollout, the ART Optimization Programme Advisory Committee, led by USAID and Unitaid and co‐chaired by WHO and the Global Fund, coordinated donor investments [23]. Meanwhile, mechanisms, such as the Antiretroviral Procurement Working Group, provided a platform for coordinating procurement and increasing transparency for suppliers [23].

Effectiveness of these collaborative platforms for information sharing is enabled through complementary and separate high‐touch, nimble coordination using bilateral and focused convenings. This is essential with CAB‐LA, as the Unitaid, WHO, UNAIDS, Global Fund and PEPFAR‐convened Coalition focuses on planning for multiple PrEP products [28]. Avoiding bottlenecks for planning that involves patented or sensitive information, such as generic licensing and incentive agreements, will require focused engagement with suppliers, payers and the negotiating party. Similarly, establishing an acceptable price for ViiV's product will require streamlined partner engagement with major purchasers. Achieving an affordable price may require innovative financing mechanisms, such as donor‐supported subsidies or volume guarantees. Negotiations to secure these interventions must commence immediately and be supported by demand forecasting, government engagement to understand potential introduction pathways and timelines, and cost transparency.

Governments, donors and major procurers will also need to collaborate closely to coordinate supply. During generic DTG rollout, catalytic procurement initiatives across early adopter countries provided a platform to coordinate efficient evidence generation and supply [23]. This will be essential in the early CAB‐LA rollout, both due to pending research questions outlined in WHO's CAB‐LA guideline [20] and ViiV's limited manufacturing capacity. In sum, effective partner coordination and alignment will require differentiated approaches, with multilateral mechanisms providing important platforms for information sharing and more targeted engagement supporting agile decision‐making and negotiation.

### 2.2 Generic development

CAB‐LA manufacture requires additional steps versus active pharmaceutical ingredient and formulation development typical for antiretroviral tablets, including particle size reduction via nanomilling, suspension formulation, sterile vial filling and gamma irradiation sterilization. While production costs for generic companies are expected to be lower than the innovator, development costs are expected to be considerably higher than more standard dosage forms [18]. Limited demand visibility is also a barrier to progressing more resource‐intensive development of a complex finished dosage form.

The Unitaid‐funded STEP‐TB project offers an example of addressing demand visibility to drive generic development. STEP‐TB market research and modelling improved understanding of the burden of tuberculosis in children and directly informed the successful introduction of dispersible, fixed‐dose combination drugs produced affordably by generic manufacturers [29]. To address similar demand visibility challenges for CAB‐LA, a need‐based demand forecast has been conducted through the Coalition to Accelerate Access to Long‐Acting PrEP. With more information on price, this model can be refined to quantify real‐life demand and inform generic production.

However, without an incentive package and appropriate market‐shaping interventions, market conditions will not support generic development at the pace or price required for rapid impact. Securing a milestone‐based incentive structure to share risks, provide accountability, and promote rapid development and commercialization of CAB‐LA is an immediate priority following the completion of licensing agreements. Supplier selection for incentives will be needed, based on detailed articulation of CAB‐LA production manufacturer capacity and capability, and potential for sustainably supporting low‐cost, quality‐assured supply. A well‐designed incentive package will also enable ceiling price negotiations using the site and process‐specific data on generic costs.

Finally, throughout development, stakeholders must support and identify the technical assistance and partnerships needed to ensure efficiency, including with ViiV, mill manufacturers and gamma irradiation suppliers.

### 2.3 National programme preparation

A fragmented landscape of over 130 post‐approval oral PrEP studies that were not linked to country priorities contributed to slow oral PrEP introduction, with a 4‐year delay between US FDA approval and earliest adoption in LMICs [30]. For example, multiple demonstration projects were conducted among female sex workers in Benin and Senegal [31, 32]. However, as of mid‐2022, neither country has continued PrEP delivery to this population [4]. In contrast, all three countries (Kenya, Nigeria and Uganda) included in the 2017 catalytic procurement initiative for DTG tablets have transitioned over 90% of adults on first‐line treatment to DTG‐based regimens [33]. These experiences demonstrate that early introduction mechanisms are unlikely to catalyse wider scale‐up without co‐ownership with government stakeholders.

To inform CAB‐LA adoption, country‐specific investment cases informed by opinion leaders, clinician societies and other stakeholders will be needed. Leveraging technical working groups, stakeholders will need to co‐develop introduction strategies and transition plans to inform M&E system development, service delivery, training and budgeting. This should be informed by readiness assessments that identify health system adaptations, considering existing infrastructure and human resources. Technical assistance will also be needed to support formative policy and guideline development.

The early introduction must address pending research questions, such as feasible testing algorithms and delivery models. Collaboration across implementation projects, including USAID's MOSAIC project, Unitaid's research in Brazil and South Africa, and other introduction studies is needed to ensure projects efficiently inform CAB‐LA scale‐up [34, 35]. Once generic CAB‐LA is available, stakeholders will need to adapt systems and approaches to drive a rapid transition to scale, leveraging existing PrEP infrastructure and progress towards de‐medicalized services. This may include capacitating SRH delivery channels by introducing PrEP diagnostic algorithms and expanding provider training. If donors and governments do not prioritize health systems preparation in parallel to other supply‐side investments, we risk another decade of small‐scale PrEP demonstration projects with limited epidemic impact.

### 2.4 Community engagement

Community networks have played an important role in demanding access to CAB‐LA and pressuring global stakeholders to prioritize meaningful, actionable consultation [36, 37, 38, 39]. Community leadership must be hardwired into all levels of global and national prevention programming to ensure that communities play a leading role in driving CAB‐LA access and person‐centred introduction planning. As seen in the early days of DTG introduction with the neural tube defect safety signal, introduction decision‐making must be led by the needs and priorities of those impacted [40].

Community advisory board platforms in the treatment space, such as those led by AfroCAB, have driven transformative new product introduction efforts for first‐ and second‐line HIV treatment options, such as TLD, paediatric DTG (pDTG) and darunavir [23]. These platforms should be adapted for prevention through engagement of populations at risk of HIV, including young people and key populations, and existing prevention advocacy networks, such as the Southern African Women Advocates. Co‐developing a community engagement strategy will be essential to informing the advocacy agenda, including micro‐grants to local networks.

Community engagement must also support sensitization and demand generation among healthcare workers and communities. To accelerate pDTG uptake, CHAI engaged community advisory board members to develop a comprehensive package of patient literacy materials, which have been adapted to local contexts [41]. Widespread access to these materials, alongside community‐led sensitization sessions with caregivers and community leaders, equipped thousands of community members with critical information, driving uptake. CAB‐LA scale‐up will require generating demand among many populations who are not already in care or taking PrEP and this will require new approaches, leveraging information channels outside facilities and health systems. To maximize the impact of community engagement, donors and governments should prioritize not only directly supporting community organizations to carry out demand‐side activities, but should also ensure that investment decision‐making is informed by the priorities of communities through meaningful consultation and co‐creation.

### 2.5 Regulatory strategy

There are several challenges associated with regulatory approval of generic CAB‐LA. First, PEPFAR's mandate around regulatory structures and pathways was developed in the context of HIV treatment. This mandate provides a critical pathway for accelerated review of ARVs for HIV treatment, allowing stringent regulatory authority review for products still under patent in the United States [42]. Without a pathway for prevention commodities, products that have not already been approved for a treatment indication must rely on WHO prequalification (PQ) and the Global Fund's Expert Review Panel. To address this challenge, stakeholders must advocate to secure support from PEPFAR and other US government partners for the revision of policies to include PrEP products, potentially involving a revised mandate or new agreement with the US FDA.

Second, while WHO PQ enables access to WHO's Collaborative Registration Procedure (CRP), which supports accelerated country reviews, achieving WHO PQ can be a bottleneck due to delayed submissions from manufacturers, lengthy review times and limited transparency on estimated timelines [43]. Moreover, although the target for WHO‐CRP review is 90 days, review times by national regulatory authorities vary widely [44]. As of April 2022, WHO PQ has included CAB‐LA on its Expression of Interest, an important first step to enable suppliers to submit for PQ [45]. Increased timeline transparency from WHO PQ will enable suppliers to prepare for commercialization and countries to prepare for procurement. An efficient, proactive strategy and coordination to secure rapid reviews and/or waivers will be required to reduce time to market in LMICs.

Finally, as a long‐acting product, CAB‐LA will require longer, more complex and more costly bioequivalence studies for regulatory submission. Generic manufacturers will need ongoing technical assistance to ensure alignment with requirements, including the design of bioequivalence studies agreed in advance with regulators or normative bodies. The US FDA has indicated its intention to release product‐specific guidance for the generic development of CAB‐LA [46]. Coordinated, proactive guidance from WHO PQ would also support efficient development by setting clear expectations for suppliers. Addressing these complex regulatory challenges for CAB‐LA will require strategic action from PEPFAR and WHO.

## 3 CONCLUSIONS

Accelerating access to affordable, generic CAB‐LA can transform progress towards HIV epidemic control. This vision of prevention impact at scale is achievable, if informed by results‐backed approaches, including those from successful product introduction in HIV treatment and family planning. VMMC also provides an important model for delivery at scale, with nearly 18 million VMMCs performed between 2016 and 2020 in 15 high‐burden African countries, driven by community engagement, innovative demand creation approaches and service delivery models (including clinic‐ and community‐based services), and a strong focus on government ownership and sustainability [47, 48].

However, CAB‐LA brings unique opportunities and challenges for HIV prevention and years of missed PrEP targets demonstrate the need to establish a new pathway, leveraging proven models from outside of HIV prevention for partnership‐driven, accelerated, impactful product introduction.

## COMPETING INTERESTS

No competing interests to declare for any author.

## AUTHORS’ CONTRIBUTIONS

SYJ, DR, AS and CA contributed to the conceptualization and outline. SYJ, DFR, ZP, MW and CA contributed to manuscript writing. All authors provided critical feedback to shape the final manuscript.

## FUNDING

United Kingdom Foreign, Commonwealth and Development Office (UK FCDO).

## Data Availability

The authors confirm that the data supporting the findings outlined in this commentary are available within the article.
